# Pretreatment Serum GDF-15 and Features of Clinical Vulnerability in Advanced Pancreatic Ductal Adenocarcinoma

**DOI:** 10.3390/jcm15207789

**Published:** 2026-10-09

**Authors:** Ji Hyun Lee, Kabsoo Shin, Tae Ho Hong, In-Ho Kim, Sook-Hee Hong, MyungAh Lee, Se Jun Park

**Affiliations:** 1 Division of Medical Oncology, Department of Internal Medicine, Eunpyeong St. Mary’s Hospital, College of Medicine, The Catholic University of Korea, Seoul 03312, Republic of Korea; jhlee0811@catholic.ac.kr; 2 Division of Medical Oncology, Department of Internal Medicine, Seoul St. Mary’s Hospital, College of Medicine, The Catholic University of Korea, Seoul 06591, Republic of Korea; 3 Cancer Research Institute, College of Medicine, The Catholic University of Korea, Seoul 06591, Republic of Korea; 4 Department of General Surgery, Seoul St. Mary’s Hospital, College of Medicine, The Catholic University of Korea, Seoul 06591, Republic of Korea

**Keywords:** growth differentiation factor 15, pancreatic ductal adenocarcinoma, prognosis, chemotherapy-related toxicity

## Abstract

**Background**: This study aimed to define the clinical significance of pretreatment serum growth differentiation factor-15 (GDF-15), including its associations with clinical vulnerability, survival, and chemotherapy outcomes in advanced pancreatic ductal adenocarcinoma (PDAC). **Methods**: Pretreatment serum samples were prospectively collected from 80 patients with locally advanced or metastatic PDAC. GDF-15 was measured using an enzyme-linked immunosorbent assay and analyzed continuously and according to the cohort median. Associations with clinicopathological characteristics and overall survival (OS) were evaluated in the overall cohort. Treatment effectiveness, delivery, and safety were additionally assessed among 68 patients who received systemic chemotherapy. **Results**: The median GDF-15 concentration was 362.6 pg/mL. Higher GDF-15 was associated with poor performance status, involvement of multiple metastatic organ sites, an elevated neutrophil-to-lymphocyte ratio, and hypoalbuminemia. Eleven of the 12 patients unable to receive chemotherapy because of clinical deterioration had high GDF-15. The high GDF-15 group had shorter OS than the low GDF-15 group in the overall cohort (median, 6.1 vs. 13.9 months; hazard ratio [HR] = 1.96; 95% CI, 1.23–3.13; *p* = 0.002). The association between continuous GDF-15 and OS was attenuated and no longer statistically significant after multivariable adjustment (HR per doubling = 1.22; 95% CI, 0.97–1.52; *p* = 0.085). Among treated patients, progression-free survival and objective response did not differ significantly between groups, whereas high GDF-15 was associated with shorter OS (median, 8.7 vs. 14.2 months; HR = 1.72; 95% CI, 1.02–2.90; *p* = 0.025) and treatment duration (median, 3.2 vs. 6.3 months; *p* = 0.007). Relative dose intensity and subsequent treatment rates were similar. Febrile neutropenia occurred exclusively in the high GDF-15 group, which also accounted for four of five treatment-related deaths. **Conclusions**: Elevated pretreatment GDF-15 was associated with limited treatment fitness and inferior survival in patients with advanced PDAC, without clear evidence of intrinsic chemotherapy resistance. GDF-15 may represent a potential marker of systemic vulnerability in advanced PDAC, although its clinical utility requires external validation.

## 1. Background

Pancreatic ductal adenocarcinoma (PDAC) remains one of the most lethal malignancies and a leading cause of cancer-related mortality worldwide [1]. In patients with advanced PDAC who are candidates for active treatment, systemic chemotherapy forms the cornerstone of disease management [2]. Modified FOLFIRINOX (mFOLFIRINOX) and gemcitabine plus nab-paclitaxel have been the principal first-line regimens for metastatic disease [3,4]. More recently, the combination of liposomal irinotecan, fluorouracil, leucovorin, and oxaliplatin (NALIRIFOX) has emerged as an additional first-line treatment option [5]. Despite advances in systemic chemotherapy, clinical outcomes remain heterogeneous and are influenced by both clinical and tumor-related factors, underscoring the need for improved risk stratification [6].

PDAC is frequently accompanied by cancer cachexia, a multifactorial syndrome characterized by ongoing skeletal muscle loss, with or without loss of adipose tissue, that leads to progressive functional impairment [7,8]. Beyond weight loss, cachexia reflects complex tumor–host interactions involving reduced food intake, systemic inflammation, and metabolic dysregulation and is associated with impaired treatment tolerance and shortened survival [7,8]. Biomarkers that capture both tumor activity and systemic vulnerability may therefore improve clinical risk assessment.

Growth differentiation factor 15 (GDF-15) has emerged as a mediator linking tumor-associated stress to systemic manifestations of cancer [9]. Normally expressed at low levels in most tissues, GDF-15 is induced by cellular stressors, including inflammation, oxidative stress, hypoxia, and oncogene activation, partly through p53-dependent signaling [10]. It regulates appetite and body weight through GDNF family receptor α-like (GFRAL)/RET signaling in the hindbrain [9]. High circulating GDF-15 has been associated with anorexia, emesis, weight loss, impaired physical function, and mortality, suggesting that it may reflect systemic vulnerability in addition to tumor burden [10,11]. In a randomized phase 2 trial that included patients with pancreatic cancer, ponsegromab, a GDF-15-neutralizing antibody, improved weight, appetite, and physical activity among patients with elevated serum GDF-15, supporting GDF-15 as a therapeutic target [12]. Beyond its metabolic effects, tumor-derived GDF-15 may promote immune exclusion by limiting T-cell trafficking, extending its relevance to cancer progression [9,13].

Although elevated GDF-15 has been associated with cachexia-related features and adverse outcomes in pancreatic cancer, its clinical implications during systemic treatment for advanced PDAC remain poorly defined [10,11]. We hypothesized that pretreatment serum GDF-15 reflects both disease burden and systemic vulnerability and could identify patients at increased risk of poor clinical outcomes. Accordingly, we examined the associations of pretreatment serum GDF-15 with clinicopathological characteristics and survival outcomes in advanced PDAC and further assessed treatment effectiveness, treatment delivery, and safety among patients who received systemic chemotherapy.

## 2. Methods

### 2.1. Patients and Samples

Between 2017 and 2021, 80 patients with advanced PDAC were enrolled at Seoul St. Mary’s Hospital, The Catholic University of Korea. Eligible patients had histologically confirmed PDAC and locally advanced or metastatic disease. Peripheral blood samples were collected at the diagnosis of advanced disease and before the initiation of first-line systemic chemotherapy, when administered. The study protocol was approved by the Institutional Review Board of Seoul St. Mary’s Hospital, The Catholic University of Korea (KC23SASI0863). All study procedures complied with the Declaration of Helsinki and applicable Korean regulations. Written informed consent was obtained from all participants before blood collection.

### 2.2. Enzyme-Linked Immunosorbent Assay (ELISA)

Peripheral blood samples were collected before the initiation of first-line palliative systemic chemotherapy. Samples were centrifuged at 2500× *g* for 10 min, and the separated serum was aliquoted and stored at −80 °C until analysis. Serum GDF-15 concentrations were measured using a commercially available Human GDF-15 DuoSet ELISA kit (DY957; R&D Systems, Minneapolis, MN, USA) according to the manufacturer’s instructions.

### 2.3. Treatment and Relative Dose Intensity

Patients considered suitable for systemic chemotherapy received first-line palliative treatment with a gemcitabine-based regimen or mFOLFIRINOX, selected at the discretion of the treating physician. Treatment was continued until radiographic or clinical disease progression or the occurrence of unacceptable toxicity. Dose reductions, treatment delays, and schedule modifications were implemented as clinically indicated to manage adverse events. Relative dose intensity (RDI) was calculated as the ratio of the delivered dose intensity to the standard planned dose intensity for the administered regimen and was expressed as a percentage. RDI was evaluated both as a continuous variable and using a cutoff of 80%.

### 2.4. Assessments and Outcomes

Tumor response was evaluated using serial radiologic imaging according to the Response Evaluation Criteria in Solid Tumors version 1.1 (RECIST 1.1). Tumor assessments were generally scheduled every 6–8 weeks and could be performed earlier when clinically indicated. Serum carbohydrate antigen 19-9 (CA 19-9) levels were measured at baseline and at the time of each tumor assessment. Progression-free survival (PFS) was defined as the interval from the initiation of first-line systemic chemotherapy to radiographic or clinical disease progression or death from any cause, whichever occurred first. Overall survival (OS) was defined as the interval from the diagnosis of advanced disease to death from any cause. Adverse events occurring during first-line chemotherapy were graded according to the National Cancer Institute Common Terminology Criteria for Adverse Events (CTCAE) version 5.0, with the highest recorded grade used for safety analysis.

### 2.5. Statistical Analysis

Continuous variables were summarized as medians with interquartile ranges (IQRs) and categorical variables as frequencies and percentages. Associations between baseline serum GDF-15 levels and clinicopathological characteristics were evaluated using the Mann–Whitney U test. Patients were classified into high and low GDF-15 groups at the cohort median. Continuous and categorical variables, including objective response rate (ORR) and disease control rate (DCR), were compared between groups using the Mann–Whitney U test and Fisher’s exact test. For selected adverse events, exploratory unadjusted risk differences (high minus low GDF-15) and 95% confidence intervals were calculated using the Newcombe–Wilson method. PFS and OS were estimated using the Kaplan–Meier method and compared using the log-rank test. The 6-month PFS and 9-month OS rates were compared using a z-test based on Greenwood standard errors. Hazard ratios (HRs) and 95% confidence intervals (CIs) were estimated using Cox proportional hazards regression. Univariable and multivariable Cox regression analyses were performed to identify factors associated with OS. Covariates for multivariable analysis were selected based on established clinical relevance. Serum GDF-15 was log_2_-transformed when analyzed as a continuous variable, such that the corresponding HR represented the change in the risk of death associated with each doubling of its concentration. The proportional hazards assumption for the multivariable model was assessed using Schoenfeld residual-based tests and diagnostic plots. Restricted cubic spline Cox models were fitted using log_2_-transformed GDF-15 and three knots at the 10th, 50th, and 90th percentiles. Likelihood ratio tests assessed overall associations with OS and nonlinearity before and after adjustment for the same covariates. All tests were two-sided, and *p* values < 0.05 were considered statistically significant. Statistical analyses were performed using IBM SPSS Statistics for Windows, version 24.0 (IBM Corp., Armonk, NY, USA); GraphPad Prism, version 11.1 (GraphPad Software, San Diego, CA, USA); Python, version 3.12.14, with the statsmodels package, version 0.15.0; and R, version 4.4.3, with the survival package, version 3.8-3.

## 3. Results

### 3.1. GDF-15 Levels in Patients with Advanced PDAC

Among the 80 patients, the median baseline serum GDF-15 concentration was 362.6 pg/mL (IQR, 260.2–618.4 pg/mL). Table 1 summarizes the clinicopathological characteristics of the study population and their associations with serum GDF-15 levels. GDF-15 levels were significantly higher in patients aged ≥ 65 years (*p* = 0.030) and those with an ECOG performance status of 2 (*p* = 0.005). Patients with metastatic involvement of ≥2 organ sites also had higher GDF-15 levels than those with involvement of 0–1 organ site (*p* = 0.008). Higher GDF-15 levels were additionally associated with an elevated baseline NLR (≥3.5; *p* = 0.003) and hypoalbuminemia (<3.5 g/dL; *p* = 0.003). No significant associations were observed with other clinicopathological characteristics, including disease status and baseline CA 19-9 levels.

### 3.2. Serum GDF-15 Levels and Prognosis

**Figure 1:**
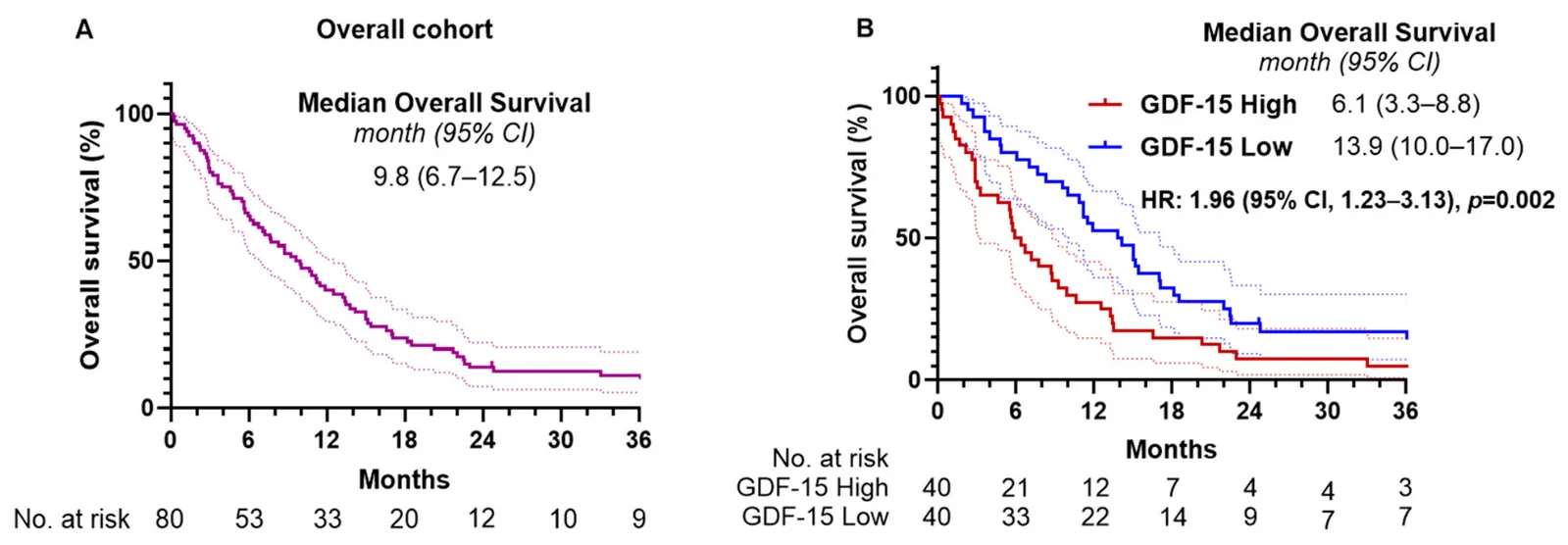
Kaplan–Meier analysis of overall survival in patients with advanced PDAC: (**A**) Overall survival in the entire study population. (**B**) Overall survival according to baseline serum GDF-15 levels, dichotomized at the median.

At a median follow-up of 66.3 months (95% CI, 66.3–73.4), 77 of 80 patients (96.3%) had died. Median OS in the overall cohort was 9.8 months (95% CI, 6.7–12.5; Figure 1A). Patients were classified into high and low GDF-15 groups based on the median baseline serum concentration of 362.6 pg/mL. The high GDF-15 group had significantly shorter OS than the low GDF-15 group (median, 6.1 vs. 13.9 months; HR = 1.96; 95% CI, 1.23–3.13; *p* = 0.002; Figure 1B). In univariable Cox regression analysis, each doubling of serum GDF-15 was associated with an increased risk of death (HR = 1.47; 95% CI, 1.21–1.79; *p* < 0.001; Table 2). This association was attenuated after multivariable adjustment and did not reach statistical significance (HR = 1.22; 95% CI, 0.97–1.52; *p* = 0.085). Older age (≥65 years; HR = 1.75; 95% CI, 1.00–3.06; *p* = 0.049) and poor performance status (ECOG PS 2; HR = 1.95; 95% CI, 1.01–3.74; *p* = 0.045) were independently associated with inferior OS. An elevated NLR (≥3.5; HR = 3.26; 95% CI, 1.85–5.69; *p* < 0.001) and markedly elevated CA 19-9 levels (≥59 × ULN; HR = 1.84; 95% CI, 1.08–3.14; *p* = 0.026) were also independent adverse prognostic factors. In exploratory spline analyses, GDF-15 was associated with OS before adjustment (*p* for overall association = 0.001) but not after multivariable adjustment (*p* = 0.213; Supplementary Figure S1). No significant evidence of nonlinearity was observed in either the unadjusted or adjusted model (*p* for nonlinearity = 0.992 and 0.642, respectively).

### 3.3. Effectiveness Outcomes in Patients Receiving Treatment

**Figure 2:**
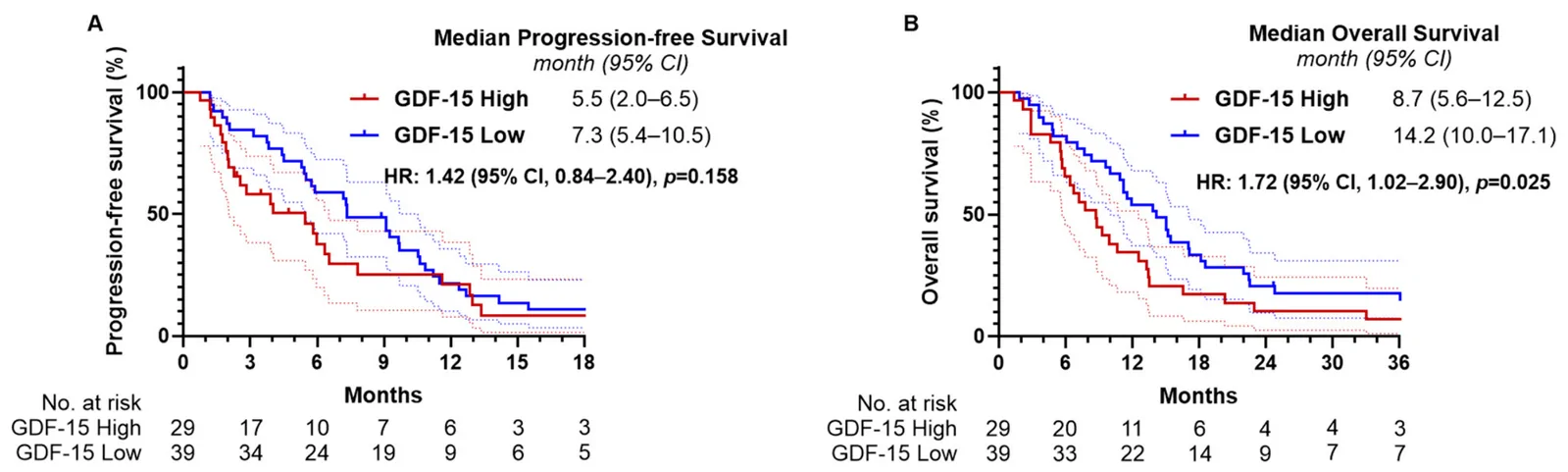
Survival outcomes according to baseline serum GDF-15 levels among patients receiving systemic therapy: (**A**) Kaplan–Meier estimates of progression-free survival and (**B**) overall survival in patients stratified by baseline serum GDF-15 level.

Of the 80 patients, 68 (85.0%) received systemic chemotherapy. Among the 12 patients who did not receive chemotherapy, 11 were in the high GDF-15 group, and only one was in the low GDF-15 group. Among treated patients, median PFS was 5.5 months (95% CI, 2.0–6.5) in the high GDF-15 group and 7.3 months (95% CI, 5.4–10.5) in the low GDF-15 group. This difference was not statistically significant (HR = 1.42; 95% CI, 0.84–2.40; *p* = 0.158; Figure 2A). In contrast, median OS was 8.7 months (95% CI, 5.6–12.5) in the high GDF-15 group and 14.2 months (95% CI, 10.0–17.1) in the low GDF-15 group. Consistent with the findings in the overall cohort, high GDF-15 levels were significantly associated with shorter OS among patients receiving systemic chemotherapy (HR = 1.72; 95% CI, 1.02–2.90; *p* = 0.025; Figure 2B).

Treatment patterns and clinical outcomes are summarized in Table 3. First-line chemotherapy regimens were similarly distributed between the high and low GDF-15 groups (*p* = 0.801). ORR was similar between the high and low GDF-15 groups (17.2% and 20.5%, respectively; *p* = 1.000), whereas DCR was numerically lower in the high GDF-15 group (58.6% and 76.9%, respectively; *p* = 0.120), corresponding to a higher proportion of patients with progressive disease as the best response in the high GDF-15 group (41.4% vs. 23.1%). The 6-month PFS rate was also numerically lower in the high GDF-15 group (37.9% and 59.0%; *p* = 0.087). Notably, the 9-month OS rate was significantly lower in the high GDF-15 group than in the low GDF-15 group (44.8% and 71.8%, respectively; *p* = 0.021).

### 3.4. Safety

All 68 patients who received systemic chemotherapy experienced at least one adverse event, and 32 patients (47.1%) experienced a grade ≥ 3 adverse event (Table 4). Grade ≥ 3 adverse events occurred in 55.2% of patients in the high GDF-15 group and 41.0% of those in the low GDF-15 group. Although grade ≥ 3 neutropenia occurred at broadly comparable rates in the high and low GDF-15 groups (51.7% and 35.9%, respectively), all six cases of febrile neutropenia occurred in the high GDF-15 group. Similarly, grade ≥ 3 thrombocytopenia and sepsis were observed exclusively in the high GDF-15 group (17.2% and 10.3%, respectively). Five treatment-related deaths occurred, including four in the high GDF-15 group and one in the low GDF-15 group. In exploratory comparisons, absolute risk differences between the high and low GDF-15 groups were 20.7 percentage points (95% CI, 6.6–38.4) for febrile neutropenia and 17.2 percentage points (95% CI, 4.1–34.5) for grade ≥ 3 thrombocytopenia. The corresponding differences were 10.3 percentage points (95% CI, −0.9 to 26.4) for grade ≥ 3 sepsis and 11.2 percentage points (95% CI, −2.2 to 28.1) for treatment-related death.

### 3.5. Treatment Exposure and Subsequent Therapy

The median treatment duration was significantly shorter in the high GDF-15 group than in the low GDF-15 group (3.2 and 6.3 months, respectively; *p* = 0.007; Table 5). Median RDI, however, was comparable between the groups (0.80 and 0.76; *p* = 0.761), as was the proportion of patients who maintained an RDI of ≥80% (51.7% and 41.0%; *p* = 0.463). Subsequent treatment was administered to 40 patients (58.8%), with similar proportions in the high and low GDF-15 groups (62.1% and 56.4%; *p* = 0.804). Among these patients, fluorouracil-based and gemcitabine-based regimens accounted for 55.0% and 40.0% of subsequent treatments, respectively.

## 4. Discussion

In this study, elevated pretreatment serum GDF-15 was associated with adverse clinical features and poorer survival in patients with advanced PDAC. Among patients receiving chemotherapy, high GDF-15 was also associated with a less favorable treatment course without a clear difference in objective response. Beyond previously reported associations with cachexia and prognosis, our study highlights treatment-related safety, with most treatment-related deaths occurring in the high GDF-15 group. Although exploratory given the limited number of events, these findings suggest that elevated GDF-15 may reflect heightened vulnerability to serious chemotherapy-related toxicity.

At baseline, higher serum GDF-15 levels were associated with older age, poor performance status, multiorgan metastases, an elevated NLR, and hypoalbuminemia. Of particular relevance, the association with an elevated NLR points to systemic inflammation, whereas hypoalbuminemia reflects inflammatory and nutritional compromise [10,14]. Collectively, these associations suggest that GDF-15 may reflect the systemic deterioration accompanying cancer cachexia and malnutrition rather than a transient inflammatory response. Conversely, GDF-15 was not significantly associated with disease status or CA 19-9 in our cohort, and a recent pancreatic cancer study similarly found no significant differences across disease stages [15]. These observations suggest that circulating GDF-15 is not a direct surrogate for tumor burden but an integrative marker of host- and disease-related stress.

Elevated GDF-15 was associated with poorer OS in univariable analysis, consistent with recent findings in PDAC [16]. Although circulating and tissue GDF-15 are distinct measures, similar associations with adverse outcomes have been reported in other cancers [17,18]. In our cohort, however, the association was attenuated and no longer statistically significant after multivariable adjustment, whereas older age, poor performance status, and an elevated NLR remained independently associated with OS. GDF-15 may partly reflect the prognostic information captured by performance status and NLR, although its incremental value remains uncertain. Unlike these indicators, however, GDF-15 may have potential as a therapeutic target. The clinical activity of GDF-15-neutralizing antibodies therefore suggests potential relevance beyond prognostic risk stratification [12,13].

Of the 12 patients unable to receive chemotherapy because of clinical deterioration, 11 had high GDF-15, suggesting an association with limited treatment fitness. Among treated patients, PFS and ORR did not differ significantly, although the high GDF-15 group had a numerically lower DCR and was more likely to have progressive disease as the best overall response, which may reflect early clinical deterioration in addition to tumor progression. Consistent with the overall cohort, high GDF-15 was associated with shorter OS and a lower 9-month survival rate among treated patients. These findings may reflect clinical vulnerability and an unfavorable treatment course. However, treatment selection may have influenced these comparisons, and the lack of significant differences in PFS and ORR does not rule out chemotherapy resistance.

Although rates of grade ≥ 3 hematologic adverse events were generally similar between groups, febrile neutropenia occurred exclusively in the high GDF-15 group. Four of five treatment-related deaths also occurred in the high GDF-15 group. These clinically consequential events may reflect reduced physiologic reserve and impaired recovery, consistent with established associations of elevated GDF-15 with cachexia and impaired physical function [10,11]. Accordingly, patients with high GDF-15 may warrant closer monitoring and proactive supportive care to mitigate the risk of serious treatment-related complications. Median RDI and the proportion achieving an RDI of ≥80% were similar between groups, whereas patients in the low GDF-15 group received treatment for substantially longer. The comparable RDI may partly reflect more frequent dose reductions and delays to manage cumulative toxicity during this longer treatment course, rather than equivalent tolerability. Despite similar rates of subsequent treatment, the high GDF-15 group had shorter OS without a significant difference in PFS. The poorer OS may partly reflect the adverse baseline clinical characteristics associated with elevated GDF-15 rather than differences in access to subsequent treatment.

Beyond these clinical associations, GDF-15 is a potentially modifiable mediator. Ponsegromab improved cachexia-related outcomes, including appetite and physical activity, in patients with cancer cachexia, supporting a functional role for GDF-15 in impaired physiologic reserve [12]. This concept is now being evaluated in the ongoing phase 2b/3 trial, which combines ponsegromab with first-line chemotherapy in cachectic metastatic PDAC [19]. Serum GDF-15 may have a role in predicting benefit from GDF-15–targeted therapy, although clinical evidence supporting its predictive value remains limited. In parallel, emerging RAS-directed therapies may offer a targeted alternative to cytotoxic chemotherapy for vulnerable patients with metastatic PDAC. Daraxonrasib improved survival and was associated with fewer grade ≥ 3 adverse events and toxicity-related discontinuations than chemotherapy [20]. These findings raise the possibility that RAS-directed therapy may carry a lower risk of severe toxicity than multiagent cytotoxic regimens. With GDF-15 inhibition potentially improving physiologic reserve and RAS-directed therapy directly targeting tumor growth, their combination may represent a therapeutic strategy worthy of prospective clinical evaluation.

This study has several limitations. Its single-center design and modest sample size limit statistical power and generalizability. The observational design and physician-directed treatment selection may have introduced residual confounding. Differences in baseline treatment fitness between treated and untreated patients may also have introduced selection bias into comparisons according to GDF-15 levels. GDF-15 was measured only once before treatment, and the cohort median was used as an internally derived cutoff without external validation. Differences in measured GDF-15 concentrations across assays may also limit the applicability of a common cutoff across studies. Dichotomization of GDF-15 also entails information loss, and the median-based group comparisons should be interpreted as exploratory. Moreover, weight loss, skeletal muscle mass, and body composition were not systematically assessed, and formal frailty, nutritional, and functional assessments were unavailable, limiting direct evaluation of clinical vulnerability, cachexia, and sarcopenia. Variation in chemotherapy regimens may have complicated the interpretation of treatment effectiveness and treatment-related adverse events. Larger multicenter studies incorporating longitudinal GDF-15 measurements and standardized nutritional and functional assessments are warranted.

## 5. Conclusions

Elevated pretreatment GDF-15 identified a clinically vulnerable subgroup of patients with advanced PDAC. These patients had limited treatment fitness and inferior survival, without clear evidence of intrinsic chemotherapy resistance. GDF-15 may therefore represent a potential marker of systemic vulnerability that could inform supportive care and the development of GDF-15-directed therapeutic strategies, although its clinical utility requires external validation.

## Figures and Tables

**Table 1:** Clinicopathological characteristics and serum growth differentiation factor-15 levels of patients with advanced pancreatic ductal adenocarcinoma.

Variables	Total (*n* = 80)	GDF-15 (pg/ml, Median, IQR)	*p* Value
**Age, Median (Range)**	67 (39–86)		
<65-year, *n* (%)	30 (37.5)	276.9 (209.3–523.7)	**0.030**
≥65-year, *n* (%)	50 (62.5)	392.3 (297.2–715.2)	
**Gender, *n* (%)**			
Male	47 (58.8)	412.6 (282.7–701.5)	0.263
Female	33 (41.2)	357.2 (240.2–522.9)	
**ECOG performance status, *n* (%)**			
0–1	64 (80.0)	351.9 (231.2–524.4)	**0.005**
2	16 (20.0)	758.3 (358.4–971.3)	
**Tumor location, *n* (%)**			
Head	34 (42.5)	431.5 (295.5–727.4)	0.153
Body/Tail	46 (57.5)	323.9 (234.4–598.0)	
**Disease status, *n* (%)**			
Locally advanced	9 (11.2)	244.5 (203.0–393.3)	0.094
Metastatic	71 (88.8)	381.5 (286.3–643.8)	
**Prior tumor resection, *n* (%)**			
No (initially advanced)	64 (80.0)	373.6 (238.3–608.8)	0.635
Yes (recurrent disease)	16 (20.0)	356.1 (308.3–679.3)	
**Number of metastatic organ sites**			
0–1	48 (60.0)	310.8 (223.0–485.5)	**0.008**
≥2	32 (40.0)	518.5 (323.0–866.0)	
**Baseline CA19-9 level, *n* (%)**			
<59 × ULN (U/mL)	53 (66.2)	348.8 (244.5–508.1)	0.080
≥59 × ULN (U/mL)	27 (33.8)	528.9 (289.7–816.2)	
**Baseline albumin, *n* (%)**			
≥3.5 g/dL	55 (68.8)	335.4 (226.1–486.7)	**0.003**
<3.5 g/dL	25 (31.2)	538.3 (311.4–947.3)	
**Baseline NLR, *n* (%)**			
≥3.5	30 (37.5)	575.6 (304.1–936.2) 342.1 (220.7–471.9)	**0.003**
<3.5	50 (62.5)		
**Site of metastatic disease, *n* (%)**			
Liver	48 (60.0)	370.4 (285.1–704.7)	0.229
Lung	15 (18.8)	365.7 (330.1–797.9)	0.128
Peritoneum	21 (26.2)	522.9 (295.5–796.4)	0.291

Bold type indicates main variable headings and statistically significant *p* values (*p* < 0.05).

**Table 2:** Univariate and multivariate analyses of the clinical features and serum GDF-15 levels for overall survival in patients with advanced pancreatic cancer.

	Overall Survival			
Variables	Univariate Analysis		Multivariate Analysis	
	HR (95% CI)	*p* Value	HR (95% CI)	*p* Value
Age ≥ 65 (vs. < 65 year)	1.65 (1.04–2.64)	0.035	1.75 (1.00–3.06)	0.049
ECOG PS 2 (vs. ECOG PS 0–1)	2.94 (1.64–5.27)	0.001	1.95 (1.01–3.74)	0.045
Metastatic disease (vs. locally advanced)	1.71 (0.82–3.59)	0.153	1.48 (0.64–3.44)	0.363
GDF-15, per doubling	1.47 (1.21–1.79)	<0.001	1.22 (0.97–1.52)	0.085
NLR ≥ 3.5 (vs. < 3.5)	2.92 (1.80–4.74)	<0.001	3.26 (1.85–5.69)	<0.001
CA 19-9 ≥ 59 × ULN (vs. < 59 × ULN)	2.01 (1.25–3.25)	0.004	1.84 (1.08–3.14)	0.026

GDF-15, growth differentiation factor-15; HR, hazard ratio; CI, confidence interval; ECOG PS, Eastern Cooperative Oncology Group Performance Status; NLR, neutrophil-to-lymphocyte ratio; CA 19-9, carbohydrate antigen 19-9; ULN, the upper limit of normal range. The HR for serum GDF-15 was calculated using log2-transformed values and represents the relative increase in the risk of death per doubling of serum GDF-15 level.

**Table 3:** Treatment outcomes according to serum GDF-15 levels in patients receiving chemotherapy.

Variables	Total (*n* = 68)	GDF-15 High (*n* = 29)	GDF-15 Low (*n* = 39)	*p* Value
**First-line chemotherapy, *n* (%)**				
Gemcitabine-based	42 (61.8)	17 (58.6)	25 (64.1)	0.801
Gemcitabine single	4 (5.9)	3 (10.3)	1 (2.6)	
Gemcitabine/Nab-paclitaxel	38 (55.9)	14 (48.3)	24 (61.5)	
mFOLFIRINOX	26 (38.2)	12 (41.4)	14 (35.9)	
**Best response, *n* (%)**				
Partial response	13 (19.1)	5 (17.2)	8 (20.5)	
Stable disease	34 (50.0)	12 (41.4)	22 (56.4)	
Progressive disease	21 (30.9)	12 (41.4)	9 (23.1)	
**Objective response rate, *n* (%)**	13 (19.1)	5 (17.2)	8 (20.5)	1.000
**Disease control rate, *n* (%)**	47 (69.1)	17 (58.6)	30 (76.9)	0.120
**Median PFS, months [95% CI]**	6.3 [5.3–9.1]	5.5 [2.0–6.5]	7.3 [5.4–10.5]	0.158
**6-month PFS, % [95% CI]**	50.2 [37.6–61.6]	37.9 [19.9–55.7]	59.0 [42.0–72.5]	0.087
**Median OS, months [95% CI]**	11.2 [8.7–13.8]	8.7 [5.6–12.5]	14.2 [10.0–17.1]	0.025
**9-month OS, % [95% CI]**	60.3 [47.7–70.8]	44.8 [26.5–61.6]	71.8 [54.9–83.3]	0.021

GDF-15, growth differentiation factor-15; mFOLFIRINOX, modified fluorouracil, leucovorin, irinotecan, and oxaliplatin; PFS, progression-free survival; OS, overall survival.

**Table 4:** Adverse events.

	Total (*n* = 68)		GDF-15 High (*n* = 29)		GDF-15 Low (*n* = 39)	
	Any Grade	Grade ≥ 3	Any Grade	Grade ≥ 3	Any Grade	Grade ≥ 3
**Any adverse event**	68 (100)	32 (47.1)	29 (100)	16 (55.2)	39 (100)	16 (41.0)
Nausea	19 (27.9)	0	9 (31.0)	0	10 (25.6)	0
Anorexia	8 (11.8)	0	4 (13.8)	0	4 (10.3)	0
Fatigue	37 (54.4)	1 (1.5)	16 (55.2)	1 (3.4)	21 (53.8)	0
Neutropenia *	39 (57.4)	29 (42.6)	19 (65.5)	15 (51.7)	20 (51.3)	14 (35.9)
Febrile neutropenia	6 (8.8)	6 (8.8)	6 (20.7)	6 (20.7)	0	0
Anemia	43 (63.2)	6 (8.8)	21 (72.4)	5 (17.2)	22 (56.4)	1 (2.6)
Thrombocytopenia	22 (32.4)	5 (7.4)	14 (48.3)	5 (17.2)	8 (20.5)	0
Infection	6 (8.8)	5 (7.4)	4 (13.8)	3 (10.3)	2 (5.1)	2 (5.1)
Sepsis	3 (4.4)	3 (4.4)	3 (10.3)	3 (10.3)	0	0
Treatment-related death	–	5 (7.4)	–	4 (13.8)	–	1 (2.6)

Data are expressed as the number of patients (%). * Includes agranulocytosis, febrile neutropenia, granulocytopenia, neutropenia, neutropenic sepsis, decreased neutrophil count, and pancytopenia.

**Table 5:** Treatment exposure and subsequent therapy according to serum GDF-15 levels.

	Total (*n* = 68)	GDF-15 High (*n* = 29)	GDF-15 Low (*n* = 39)	*p* Value
**Duration of treatment, months**	4.7 (1.6–7.2)	3.2 (1.4–5.0)	6.3 (4.2–8.0)	0.007
**Relative dose intensity**	0.78 (0.64–0.89)	0.80 (0.65–0.89)	0.76 (0.63–0.89)	0.761
**RDI ≥ 80%**	31 (45.6)	15 (51.7)	16 (41.0)	0.463
Subsequent treatment *	40 (58.8)	18 (62.1)	22 (56.4)	0.804
Fluorouracil-based	22 (55.0)	8 (44.4)	14 (63.6)	
Gemcitabine-based	16 (40.0)	8 (44.4)	8 (36.4)	
Others	2 (5.0)	2 (11.1)	0	

GDF-15, growth differentiation factor-15; RDI, relative dose intensity. Data are expressed as *n* (%) or median (IQR). * Percentages for subsequent treatment regimens were calculated among patients who received second-line treatment.

## Data Availability

The datasets used in the current study are available from the corresponding author upon request.

## Supplementary Materials

The following supporting information can be downloaded at: https://www.mdpi.com/article/10.3390/jcm15207789/s1, Supplementary Figure S1. Restricted cubic spline analysis of pretreatment serum GDF-15 and overall survival.
