# Supplementary material for: Pretreatment Serum GDF-15 and Features of Clinical Vulnerability in Advanced Pancreatic Ductal Adenocarcinoma
Source: J Clin Med. 2026 Oct 9;15(20):7789. doi: 10.3390/jcm15207789 (PMC13704926; doi:10.3390/jcm15207789)
Supplement: Supplementary file 1 [file jcm-15-07789-s001.zip › jcm-4562472-supplementary.pdf]

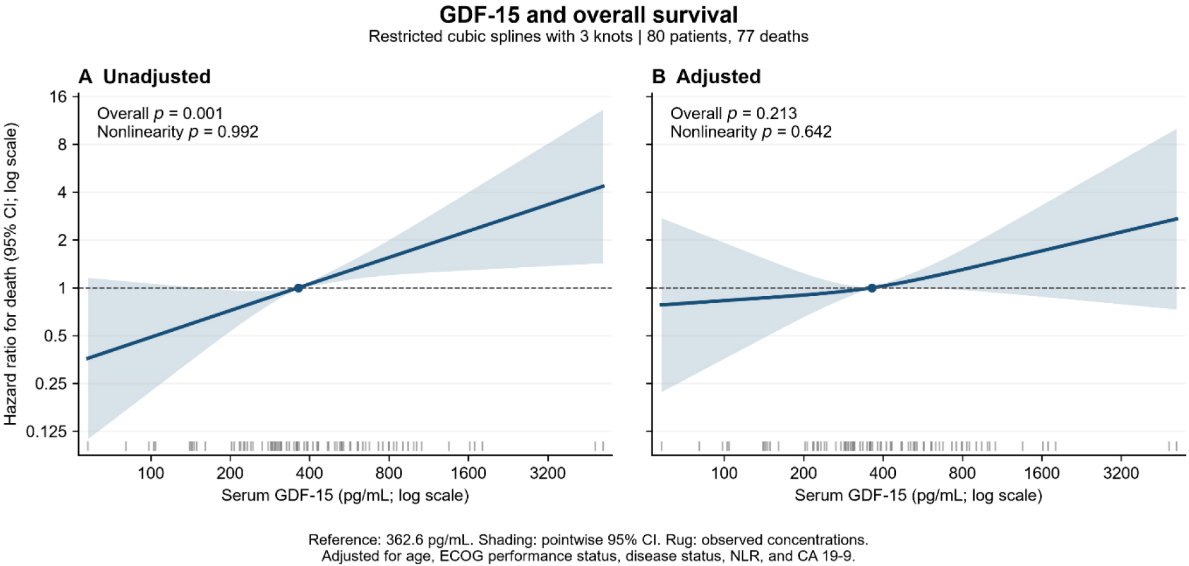

Supplementary Figure S1. Restricted cubic spline analysis of pretreatment serum GDF-15 and overall survival: (A) unadjusted and (B) multivariable-adjusted associations. Solid lines represent hazard ratios, and shaded areas indicate 95% confidence intervals, using the cohort median GDF-15 concentration (362.6 pg/mL) as the reference. Models used three knots at the 10th, 50th, and 90th percentiles of log_2-transformed GDF-15. The adjusted model included age, ECOG performance status, disease status, NLR, and CA 19-9.
